# Soil legacy determines arbuscular mycorrhizal spore bank and plant performance in the low Arctic

**DOI:** 10.1007/s00572-020-00977-5

**Published:** 2020-07-29

**Authors:** Minna-Maarit Kytöviita, Mauritz Vestberg

**Affiliations:** 1 Department of Biological and Environmental Science, University of Jyvaskyla, 40014 Jyvaskyla, Finland; 2 Natural Resources Institute Finland (Luke), Helsinki, Finland

**Keywords:** AM spore bank, Acaulosporaceae, Monoculturing, *Solidago virgaurea*

## Abstract

Human impact is rapidly changing vegetation globally. The effect of plant cover that no longer exists in a site may still affect the development of future vegetation. We focused on a little studied factor—arbuscular mycorrhizal (AM) fungus spore bank—and its effect on three test plant species. In a low Arctic field site, plots were maintained for 6 years, devoid of any vegetation or with a *Solidago virgaurea* monoculture cover. We analysed the AM fungal morphospecies composition and identified 21 morphospecies in the field plots. The AM morphospecies community was dominated by members of Acaulosporaceae. Monoculturing under low Arctic field conditions changed the soil AM spore community, which became dominated by *Glomus hoi.* We tested the soil feedback in the greenhouse and grew *Solidago virgaurea*, *Potentilla crantzii* and *Anthoxanthum odoratum* in the field soils from the plots without plant cover, covered with *Solidago virgaurea* or with intact vegetation. Our results suggest that monoculturing resulted in improved N acquisition by the monocultured plant species *Solidago virgaurea* which may be related to the AM fungus community. Our results show that a rich community of AM fungus spores may remain viable under field conditions for 6 years in the low Arctic. Spore longevity in field soil in the absence of any host plants differed among AM fungus species. We suggest that AM fungus spore longevity be considered an AM fungal life-history trait.

## Introduction

Soil legacy is the persistent effect of previous land use or vegetation on soil properties such as species richness, productivity and resilience. Land use effects on arbuscular mycorrhizal (AM) fungal communities are interesting because of the importance of AM fungi in ecosystem functions. Most plants engage in AM symbiosis (Brundrett 2002). Generally, the AM fungus acquires soil nutrients which are exchanged for host plant carbon at symbiosis-specific structures called arbuscules (Smith and Read 2017). AM fungi improve the acquisition of growth-limiting mineral nutrients such as nitrogen (Govindarajulu et al. 2005; Whiteside et al. 2012), phosphorus (Smith et al. 2011) and potassium, in particular (Garcia and Zimmermann 2014). Human activities such as pasturing and growing crops affect AM fungal community composition (e.g. Cofré et al. 2017; Oehl et al. 2010). However, there seems to be no consensus on the ecological importance of land management effects on AM fungus species (Jansa et al. 2014). Furthermore, there is very little information on AM fungus species in Arctic ecosystems and their response to environmental changes.

When plant species grow in monoculture, a specific fungal (Becklin et al. 2012) and bacterial (Bulgarelli et al. 2015) microbial community may be selected consequently. Soil communities have generally negative feedback to the monoculture host plant (Klironomos 2002), and the phenomenon is well-known in agriculture where repeatedly cultivated monocultures of crops result in declining harvest yields (Hennessy 2006). Heterospecific effects are less known but may differ between plant functional groups (Kos et al. 2015). In both hetero- and conspecific cases, plant-associated microbes are an important determinant of plant performance and subsequent plant community composition (Olff et al. 2000; Koziol and Bever 2017).

The estimated number of AM fungal taxa varies, but based on spore morphology, 334 AM fungal species have been identified (amf-phylogeny.com/amphylo_species.html, accessed 13.3.2020). In any one location, the AM fungal morphospecies richness rarely exceeds 30 (Jansa et al. 2002; Oehl et al. 2003; Dumbrell et al. 2010a; Kivlin et al. 2011). The composition of the AM fungal community has been shown to affect the host plant community composition (e.g. Gange et al. 1993; Bever 2003; Stampe and Daehler 2003). However, the reverse is also true: host plant community affects the fungal community (Burrows and Pfleger 2002; Johnson et al. 2003). Host and AM fungal relationships depend on the identities of partners in the sense that the plant benefit of symbiosis is constrained by the fungal identity and the fungal benefit, defined for instance as the amount of spores produced, depends on the host plant identity (Sanders and Fitter 1992; Bever et al. 1996; Eom et al. 2000). The relationship is complex, and between a given plant and fungus, the benefit to the partners is affected by the presence of alternative symbionts for both partners (Pietikäinen et al. 2007; Pearson et al. 1993). Soil characteristics and plant community partially regulate the soil AM fungus spore community, and spore communities are different in different vegetation types (Velázquez et al. 2013). However, as AM spores are long lasting, it is not only the current plant community but also the past plant communities that may partially determine AM spore communities in a given soil. In this way, past and present vegetation and AM fungal community affect future plant performance.

Arctic AM fungal communities and their roles in the ecosystem are poorly known. There is no report of any AM fungal genera or species specific to cold climates; instead, one family, the Gigasporaceae, is reported to be absent from cold soil (Walker 1992; but see Stürmer et al. 2018). AM function in Arctic soils could be hampered because of the cold soil temperature and short growing season (Kytöviita 2005). Nevertheless, low Arctic plants are commonly mycorrhizal (Pietikäinen et al. 2005), and some members of the mycorrhizal plant families may have high AM fungal colonisation levels even in the high Arctic (Olsson et al. 2011). In greenhouse tests, AM symbiosis increases nutrient acquisition and growth of low Arctic herbs (Kytöviita et al. 2003; Kytöviita and Ruotsalainen 2007), suggesting that arbuscular mycorrhizas are an important part of Arctic plant ecology.

In the present study, we explored the AM fungal morphospecies community in a low Arctic field in (1) natural meadow, (2) plots maintained with *Solidago virgaurea* monoculture cover for 6 years and (3) plots maintained for 6 years devoid of any vegetation. With this experimental set-up, we answered the following research questions: (i) What is the AM fungal morphospecies composition and richness in natural low Arctic meadow? (ii) How diverse is the AM fungal community maintained by monoculturing one host plant species? (iii) Do AM fungal spores form a persistent spore bank under field conditions? (iv) What is the soil-mediated effect of loss of vegetation and monoculturing on future plant performance.?

We attempted to identify ecological interactions between AM fungi and host plants by exploring AM fungal linkages in terms of correlation of AM morphospecies abundance in vegetated plots. The AM morphospecies community in plots without vegetation represents the persistent AM fungal spore bank. Furthermore, we tested soil feedback in the greenhouse using three different low Arctic plants including the original field monoculture species. Based on previous reports (Bever 2002; Mangan et al. 2010), we expected *Solidago* grown in the *Solidago* monoculture history soil to perform relatively poorly in comparison to other plant species. To link the soil treatments in the field with plant and fungal responses in the greenhouse, we measured AM fungal root responses to the three treatments. Moreover, we measured soil OM and soluble and microbial N pools as proxies of soil nutrient availability, and linked that to plant N acquisition. We focused on N because of its pivotal role in Arctic ecosystems.

## Materials and methods

### Field experiment

We selected two low Arctic south-facing meadows at about 600 m above sea level with similar vegetation located about 2.5 km apart in two separate valleys in the Kilpisjärvi area, NW Finland (69°03′ N, 20°50′ E and 69°05′ N, 20°47′ E). Both sites are in the summer grazing area of reindeer, and the history of reindeer herding in this area dates back at least a few centuries. The vegetation of these sites is relatively rich with about 30 common vascular plant species. The sites are dominated by the grass *Deschampsia flexuosa*, but sedges and herbs, such as *Solidago virgaurea*, *Trollius europaeus* and *Bistorta vivipara*, also are frequent. Only a few species of dwarf shrubs such as *Betula nana* and *Vaccinium myrtillus* occur. The vegetation is described in detail in Pietikäinen et al. (2007). The length of the growing season is about 90 days in these meadows, and the mean annual temperature is − 2.56 °C (1951–1985) and precipitation 422 mm (1961–1985) measured at Kilpisjärvi meteorological station situated at 483 m a.s.l. (Järvinen 1987). The average soil temperature at 3–5 cm depth during the snow-free months (July and August) is 10.8 °C (2000–2006; Kytöviita, unpublished). The soil chemistry in response to the treatments (see below) is detailed in Kytöviita et al. (2011).

At the end of June 1999, nine experimental plots 3.5 m in diameter were established on each site. The distance between plots within a site was between 1 and 42 m. The plots were randomly allocated to three treatments: (i) intact meadow as a reference ‘control’, (ii) *Solidago* monoculture and (iii) no-plants. Thus, there were three replicate plots for each treatment per site totalling 18 plots. All the vegetation was removed from the monoculture and the no-vegetation plots by hand and the peripheries of all the plots were trenched to a depth of 25 cm. The trenches were renewed annually. Most roots were extracted, but small fragments of fine roots could not be completely removed. At this stage, these manipulations left the no-vegetation and the monoculture plots without any aboveground vegetation. The monoculture plots were revegetated in August 1999 by planting 100 mature *Solidago virgaurea* plants in each plot. The plant material for revegetation was collected from the surrounding undisturbed meadow. We chose *Solidago* as the monoculture species because it is common in a wide range of habitats. The control plots were left unmanipulated presenting the diversity of natural vegetation. All the plots were covered with light (weight 17 g m^−2^) transparent white mesh every year from mid-August to early June to prevent the natural seed rain and subsequent seedling establishment. We could not detect any effect of the mesh on soil properties or soil temperature (Mikola et al. 2014). The few seedlings emerging from the seedbank in the monoculture and no-plant plots were hand-weeded annually. The plots were fenced to exclude reindeer.

### Arbuscular mycorrhizal spore community

In September 2005, twelve soil cores (3 cm diameter × 6 cm depth) were taken from each plot and the AM spores were extracted from a 160 g subsample of the sieved (4 mm) and thoroughly mixed soil of each plot. Spores belonging to the phylum Glomeromycota were extracted by wet sieving and decanting (Gerdemann and Nicolson 1963) followed by centrifugation in water and in a 50% sucrose solution (Walker et al. 1982). A 500-μm and a 50-μm sieve were used for wet sieving. On the larger sieve, sporocarps and spores tightly adhering to roots were found. After centrifugation, spores were washed into a Petri dish for examination under a dissecting microscope at magnifications up to × 50 with illumination by incident light from a fibre-optic, quartz-halogen light source with a colour temperature of 3200 K (Walker et al. 1993). Spores were counted and, whenever possible, identified to species using a high-power light microscope. Spore morphology studies were carried out by examining the features of spores mounted in polyvinyl-lactoglycerol amended with Melzer reagent (Omar et al. 1979) when necessary. Spores were identified to genus and species by using various websites, for example the INVAM site (https://invam.wvu.edu/). Identification was also done from original species descriptions, of which most can be downloaded from the site www.amf-phylogeny.com. Endurance% estimates of the persistence rates of AM fungus spores in the soil were calculated as the abundance of spores discovered in no-plant soil relative to control soil multiplied by 100. The maximum value was set to 100.

### Greenhouse experiment

We evaluated the effects of the treatments applied in the field on plant performance in the greenhouse. To do that, three test plant species were grown separately in composited soil samples from one field plot. There were three treatments (control intact meadow, *Solidago* monoculture and no-plant) each with six replicate field plots, thus 18 field plots in total. Soil from these tested three plant species resulted in 54 pots in the greenhouse experiment. The test species were *Anthoxanthum odoratum* (Poaceae; *Anthoxanthum* hereafter), *Potentilla crantzii* (Rosaceae; *Potentilla* hereafter) and *Solidago virgaurea* (Asteraceae; *Solidago* hereafter). They are common in low Arctic meadows and all are reported to form arbuscular mycorrhizas (Read and Haselwandter 1981; Eriksen et al. 2002). Seeds of the test plant species were collected near the experimental field plots at Kilpisjärvi in September 2004. The seeds were kept in moist sterilised sand at 5 °C until use in the greenhouse experiment on 20 July 2005. The seeds germinated within 15 days.

In August 2005, 15 soil cores (3 cm diameter × 6 cm depth) were collected from each no-plant and monoculture plot. In each control plot, 20 soil cores were collected because the removal of roots reduced the volume of soil usable for the experiment. The 15 soil cores per plot (20 in the case of the control treatment) were mixed and sieved (4 mm) and pots (8 × 8 × 8 cm) were filled with the mixed soil. As a result, in each single pot, there was soil composited from one field plot. On 26 August 2005, one seedling was planted in each pot. The number of replicates was 6 per soil treatment and species. Supplemental light was provided by 400-W Osram HQI lamps for a photoperiod of 20-h light and 4-h darkness simulating Arctic conditions prevailing during the growing season of the plants. The plants were grown in the Oulu University botanical garden greenhouse experimental unit for 5 months. The position of the pots was random on a table, and the pots were re-randomised every 2 weeks. The pots were watered with tap water when necessary, but not fertilised.

At termination of the experiment, plant shoots were separated by cutting, and roots were gently separated from the soil. A soil sample for N analyses was carefully taken from each pot and stored at 5 °C until analysed within 2 weeks. The roots were washed with copious water, and then fresh weights of the total root system and of 10 root fragments were measured; the 10 root fragments were stored in 50% ethanol, and the rest of the root system was oven-dried together with the shoots (60 °C, 48 h).

### Soil and plant analyses

Moisture (105 °C, 12 h) and organic matter (OM) contents (determined by ashing at 475 °C for 4 h; expressed as percentage OM per dry weight soil) were gravimetrically determined from soil samples at the end of the greenhouse experiment. A subsample of ca. 10 g fresh soil was extracted with 50 mL of 0.5 M K_2_SO_4_, and oxidation of the total extractable nitrogen compounds into nitrate–nitrogen was carried out by the peroxodisulfate (K_2_S_2_O_8_) oxidation method. The NO_3_-N concentration in the extracts was determined with a Lachat Autoanalyser (Lachat Instruments, Milwaukee, WI, USA). Microbial N was extracted from the samples using 0.5 M K_2_SO_4_ after chloroform fumigation (48 h) (Brookes et al. 1985) and was analysed as the total extractable N after oxidation as above. Microbial N was calculated by subtracting total extractable N in the non-fumigated extracts from that in the fumigated ones.

Shoot N concentration ([N]) was analysed using the dynamic flash combustion technique (EA 1110 Elemental Analyser, CE Instruments, Wigan, UK). We measured nitrogen because it is considered the commonly growth-limiting nutrient in the Arctic (Chapin III et al. 2011), and AM fungi are known to transfer N to host plants (e.g. Whiteside et al. 2012). The root fungal colonisation intensities in the stored root fragments were assessed using a modified (no phenol) trypan blue staining method of Phillips and Hayman (1970) and the gridline intersection method of McGonigle et al. (1990). Ten intersections with each of the ten root fragments per plant were scored. For each intersection, we scored the presence of classic blue staining arbuscular mycorrhizal structures (hyphae, arbuscules and vesicles).

### Statistical analyses

Field data on AM fungal community composition differences were analysed with PERMANOVA. Data on AM fungal abundance, morphospecies richness and diversity were analysed by one-factor analysis of variance (ANOVA; factor: soil treatment). The Shannon-Weiner index was calculated as a measure of AM morphospecies diversity. The index combines two components of diversity, i.e. species richness and evenness. The assumptions of normal distribution and homogeneity of variances were checked with the Kolmogorov-Smirnov test and Levene’s test. AM morphospecies co-occurrence in the field plots was analysed with Pearson correlation analyses. Field data of the four most common AM morphospecies was reduced to one principal component in a principal components analysis. The first principal component (PC-1) explained 55% of the variation in spore abundance.

Greenhouse experiment data on plant parameters (plant biomass and N content) were assessed with analysis of covariance (ANCOVA) where the plant species tested was included as a fixed factor, and PC-1 (reflecting the AM fungal community) and soil organic matter were included as covariates. The frequency of fungal structures in plant roots and soil OM and soil microbial N were analysed by two-factor ANOVA (factors: plant species and soil history). Correlations between fungal structure frequencies in plant roots, soil soluble N, soil microbial N, plant N content and N concentration were assessed by Spearman’s non-parametric correlation analyses. Correlations between the spore abundances of AM taxa were assessed using Pearson correlation analyses.

All analyses were carried out with SPSS version 24.0 except the spore network and PERMANOVA which were performed with R 3.5.1.

## Results

### Field experiment

**Fig. 1:**
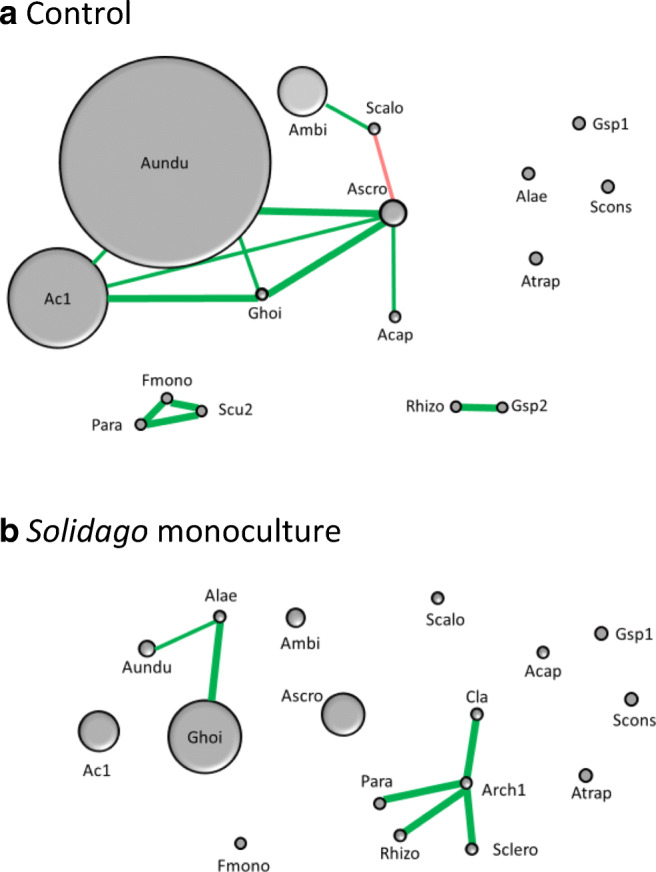
Correlation network of arbuscular mycorrhizal (AM) spore abundance in the intact control soil (**a**) and after monoculturing *Solidago virgaurea* for 6 years in low Arctic meadow (**b**). The AM taxa are Acap = *Acaulospora capsicula*, Alae = *A. laevis*, Ascro = *A. scrobiculata*, Aundu = *A. undulata*, Ac1 = *Acaulospora* sp. 1, Ac2 = *Acaulospora* sp. 2, Ambi = *Ambispora fennica*, Atrap = *Archaeospora trappei*, Cla = *Claroideoglomus claroideum*, Fun = *Funneliformis monosporus*, Ghoi *Glomus hoi*, Gsp1 = *Glomus* sp. 1, Gsp2 = *Glomus* sp. 2, Para = *Paraglomus occultum*, Rhizo = *Rhizophagus intraradices*, Scalo = *Scutellospora calospora*, Sclero = *Sclerocystis rubiforme*, Scu2 = *Scutellospora* sp. 2, Scon = *Septoglomus constrictum*. Only statistically significant positive (green) or negative (red) correlations are shown; thick lines *p* ≤ 0.01, thin lines *p* ≤ 0.05. The size of the symbol is relative to the abundance of the most common species *Acaulospora undulata* in the control treatment soil. In case of rare species, the symbol size was fixed to allow visibility and therefore not in scale

**Fig. 2:**
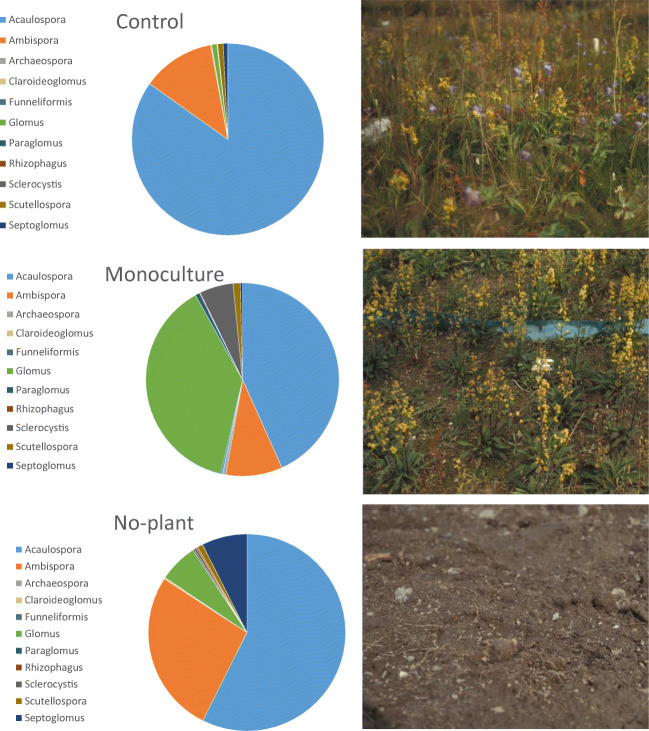
Arbuscular mycorrhizal fungal spore community composition in low Arctic intact field soil with natural vegetation (control), after 6 years of monoculturing the host plant *Solidago virgaurea* (monoculture) and after 6 years without any plant cover (no-plant). The pie slices are relative spore abundances of the AM genera in 100-g field soil

The two sites differed in the total number of spores: one site had on average 730 ± 197 spores per 100-g soil and the other 259 ± 49 spores in the intact meadow. The two sites differed also in terms of the AM morphospecies community composition (PERMANOVA *R*^2^ = 0.15, *p* = 0.026), but the common AM taxa were the same. The AM community responded to the treatments in a similar manner (PERMANOVA site × soil history interaction *R*^2^ = 0.03, *p* = 0.965), and the sites did not differ in AM morphospecies diversity. Therefore, average values per treatments are shown in Table 1 and in Figs. 1 and 2.

**Table 1:** The different AM fungal species and types detected per 100-g dry weight soil

Glomeromycota species and types	Control	No-plant	Monoculture	Endurance%		
*Acaulospora capsicula*	0.8	0	2.8 (1%)	0		
*Acaulospora laevis*	1.0	1.2 (2%)	0.3	100		
*Acaulospora scrobiculata*	32.5 (7%)	5.8 (7%)	51.4 (21%)	18		
*Acaulospora undulata*	262.5 (53%)	15.7 (20%)	19.3 (8%)	6		
*Acaulospora* sp. 1, small, red-brown, ornamented	122.6 (25%)	22.2 (28%)	45.8 (19%)	18		
*Ambispora fennica*	60.7 (35%)	20.9 (26%)	21.3 (9%)	35		
*Archaeospora trappei*	0.7	0.6	1.1	86		
*Archaeospora* sp. 1, very small	0	0	0.2			
*Acaulospora* sp. 2, small, red-brown, smooth	0	0.1	0	100		
*Claroideoglomus claroideum*	0	0.3	0.3	100		
*Funneliformis monosporum*	0.7	0	0.7	0		
*Glomus hoi*	3.3	4.5 (6%)	85.4 (35%)	100		
*Glomus* sp. 1., dark brown, loose sporocarp	0.8	0.4	0.5	50		
*Glomus* sp. 2, small, hyaline	0.1	0.1	0	100		
*Paraglomus occultum*	0.2	0.3	1.7	100		
*Rhizophagus intraradices*	0.4	0	0.3	0		
*Sclerocystis rubiforme*	0	0.3	12.7 (5%)	100		
*Scutellospora calospora*	4.4	0.6	2.7 (1%)	14		
*Scutellospora* sp. 1, middle-sized, dark brown, ornamented	0	0.1	0	100		
*Scutellospora* sp. 2., middle-sized, shiny, irregular	0.2	0	0	0		
*Septoglomus constrictum*	3.8	5.8 (7%)	0.9	100		
Total number of spores in 100 g soil	494 ± 139a	79 ± 13b	225 ± 46a			
Species number in 100 g soil	9 ± 0.9	9 ± 0.7	9.5 ± 0.3			
Diversity SWI	1.24 ± 0.09	1.48 ± 0.05	1.44 ± 0.07			

In the field, plots were maintained for 6 years without plant cover (no-plant), with *Solidago virgaurea* monoculture (monoculture) or without manipulations (control), *n* = 6. In parentheses, the relative abundance > 1% of the taxa within treatment is given. Endurance% estimates the persistence rate of the AM spores in the field soil*. SWI* Shannon-Weiner diversity index

In total, 21 morphospecies belonging to 11 genera were detected in the field soil (Figs. 1 and 2; Table 1). However, only four species were common (*Acaulospora scrobiculata, A. undulata, Acaulospora sp. 1, Ambispora fennica*) and discovered in at least 17 of the 18 plots. Four species were discovered only in one plot (two *Scutellospora* species, one *Archaeospora* species and one *Acaulospora* species that could not be resolved to species level). Altogether, the fungal morphospecies population was dominated by members of Acaulosporaceae. In an attempt to discern ecological relationships within the potentially active AM fungal community, we explored the AM spore community with Pearson’s pairwise correlation analysis in the vegetated plots (Fig. 1). The resulting spore abundance networks are characterised by many positive interactions in the unmanipulated control treatment and complex network structure (Fig. 1a). In contrast, the networks in the monoculture plots are short and do not connect the dominant species (Fig. 1b).

AM spores were more abundant in intact control soil than in the monoculture soil or the no-plant soil, but the difference was not statistically significant between control and monoculture soils (Table 1). Persistence in the soil spore bank was species-specific (Table 1). Although the soil history affected AM morphospecies community composition (PERMANOVA *R*^2^ = 0.33, *p* = 0.001), the soil history did not affect AM morphospecies richness or diversity significantly (Table 1). According to variation in the PC-1 loadings followed by ANOVA and Tukey’s multiple range test, the control soil spore community composed of the four most common species was significantly different from the no-plant and monoculture soils (*F*_(2,17)_ = 14.1, *p* < 0.01). This was due to the high abundance of members of Acaulosporaceae in the control soil (Fig. 2).

### Feedback to the plants

**Fig. 3:**
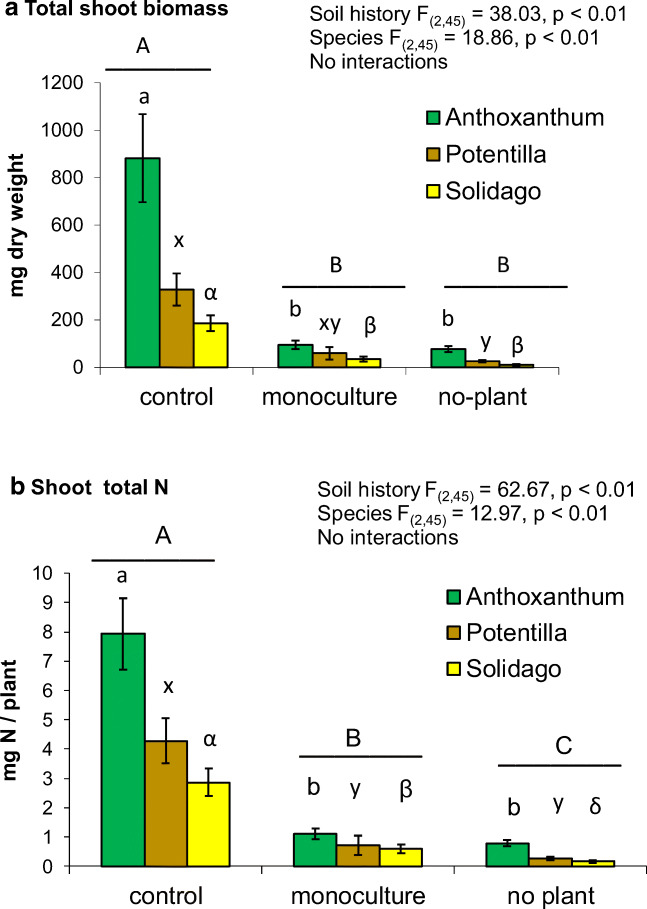
**a** Total plant biomass (mg dry weight per plant) and **b** total N capture (mg N per plant) in the *Anthoxanthum odoratum*, *Potentilla crantzii* and *Solidago virgaurea* plants grown in the greenhouse in field soil from unmanipulated control, *Solidago* monoculture and the no-plant treatments. Mean values ± SE are shown. *F* statistics refer to two-factor ANOVA. Different letters among soil histories (above horizontal lines) and within plant species denote significant differences according to Tukey’s multiple range test

Plant growth and total N capture were significantly different in the soils with different histories: all plants were able to acquire significantly more biomass when grown in control than in the no-plant soil (Fig. 3a). *Solidago* N capture was higher from *Solidago* monoculture soil in comparison to no-plant soil, whereas monoculturing did not improve N acquisition by *Potentilla* and *Anthoxanthum* (Fig. 3b) significantly.

Soil OM % was significantly lower in the monoculture (3.9 ± 0.7%) and no-plant plots (4.3 ± 0.5%) in comparison to intact meadow (9.3 ± 1.3%) (*F*_2,15_ = 8.75, *p* < 0.01). Because the treatments affected soil OM and fungal population (Table 1), their role in N acquisition by the three plant species was investigated with ANCOVA with soil OM and the fungal community (PC component 1) as covariates for the three treatments separately (Table 2). These results showed that, in the control and no-plant soil, plant species, soil OM and AM fungal community significantly affected plant growth (Table 2). In the control soil, only soil OM explained plant total N acquisition (Table 2). In the no-plant soil, only plant species was significant in terms of plant total N acquisition (Table 2). In the monoculture soil, both AM fungal community and soil OM explained a significant proportion of the variation in plant N acquisition with marginal effects by the plant species (Table 2).

**Table 2:** ANCOVA results on effects of plant species (plant), AM spore community (AM) and soil organic matter (soil OM) on plant biomass and total N content in the (A) control soil, (B) *Solidago* monoculture soil and (C) no-plant soil

		Biomass		N content	
	*df*	*F*	*p*	*F*	*p*
(A) Control soil					
Plant	2	19.892	< 0.01	0.696	0.139
AM	1	12.196	< 0.01	2.650	0.129
Soil OM	1	9.027	0.01	4.837	0.048
(B) *Solidago* monoculture soil					
Plant	2	3.561	0.058	3.273	0.071
AM	1	7.002	0.020	15.579	< 0.01
Soil OM	1	1.075	0.319	5.814	0.031
(C) No-plant soil					
Plant	2	16.192	< 0.01	19.504	< 0.01
AM	1	9.493	< 0.01	2.595	0.131
Soil OM	1	12.260	< 0.01	2.258	0.157

The plant species were *Anthoxanthum odoratum*, *Potentilla crantzii* and *Solidago virgaurea*; AM spore community was reduced to one principal component (PC-1). Soil OM and PC-1 were included as covariates in the model

**Fig. 4:**
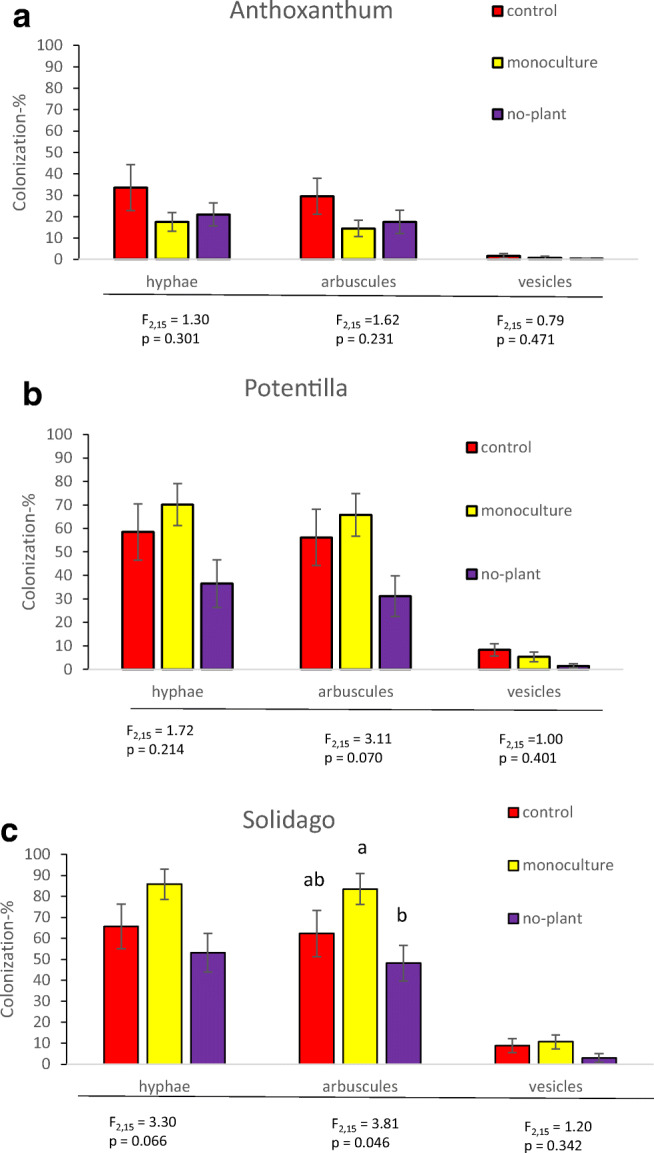
Colonisation intensity of arbuscular mycorrhizal structures (hyphae, arbuscules and vesicles) in the roots of **a***Anthoxanthum odoratum*, **b***Potentilla crantzii* and **c***Solidago virgaurea* when grown under greenhouse conditions in field soil from unmanipulated control, *Solidago* monoculture and the no-plant treatments. Mean values ± SE are shown. One-factor ANOVA results are shown at the bottom of the graphs. Only significantly different means are marked; those marked with the same letter do not differ significantly according to Tukey’s test, *p* < 0.05

The soil microbial N was significantly higher in the control soil (135 ± 5 μg N g^−1^ soil) than in the monoculture (40 ± 3 μg N g^−1^ soil) or no-plant soil (35 ± 4 μg N g^−1^ in soil) at the end of the greenhouse experiment (*F*_2,15_ = 56.84, *p* < 0.01). The plant species had no effect on soil microbial N. Despite the differences in microbial N pools, the soil soluble N was not significantly different between the treatments. Soil soluble N and plant shoot [N] correlated significantly with the frequency of mycorrhizal structures in plant roots (Table 3). Of the three plant species, only *Solidago* root fungal frequency responded statistically significantly to the treatments. The frequency of arbuscules was significantly higher in the monoculture soil in comparison with that in the no-plant soil (Fig. 4).

**Table 3:** Relationship between soil soluble N (N soluble), soil microbial N (Nmicr), plant shoot concentration (shoot [N]), plant total content (total N) and frequency of AM fungal structures hyphae, arbuscules and vesicles in roots

	Nmicr	shoot [N]	Total N	Hyphae	Arbuscules	Vesicles	
N soluble	0.100 0.470	0.279 *0.043*	0.024 0.866	0.427 *0.001*	0.429 *0.001*	0.404 *0.002*	
Nmicr		0.152 0.277	0.662 *0.001*	0.221 0.109	0.235 0.087	0.323 *0.017*	
Shoot [N]			− 0.120 0.392	0.510 *0.001*	0.519 *0.001*	0.443 *0.001*	
Total N				− 0.077 0.583	− 0.060 0.672	0.179 0.199	
Hyphae					0.993 *0.001*	0.679 *0.001*	
Arbuscules						0.673 *0.001*	

Correlation coefficients (Spearman’s rho) followed by 2-tailed significances are shown, significant *p* values (*p* < 0.05) are italicized, *n* = 53–54

## Discussion

### AM species richness

In total, 21 AM fungal morphospecies belonging to 11 genera were detected in the field soil. This indicates a high richness at high taxonomic levels in the Arctic meadows. In comparison, the same number of genera was discovered when exploring 16 sites across a range of different soils in Central Europe (Oehl et al. 2010). The AM fungal species richness estimates based on spore morphology range between15 and 35 in temperate systems (Oehl et al. 2003; Vestberg et al. 2011) and the discovery of 21 AM fungal morphospecies in the present low Arctic meadow plots is surprisingly high. We first sampled 24 × 80 g soil and identified 17 morphospecies, and after doubling our sampling, a further four morphospecies were discovered. Consequently, it seems likely that with more extensive sampling, more AM fungal species would have been discovered and that the true number of AM fungal species in these low Arctic meadows is higher than 21. The relatively high number of AM fungal species is comparable to 23 morphospecies described in a high-altitude meadow in Tibet (Gai et al. 2009) supporting the view that AM fungi are truly diverse in cold climate habitats.

Factors affecting AM fungal richness are poorly known. Generally, species richness declines with increasing latitude (Hillebrand 2004). Therefore, we expected the number of AM fungal taxa to be lower in the present low Arctic habitat than in temperate and tropical habitats if the AM fungi follow the same trend as aboveground organisms. It has been suggested that host plant diversity regulates symbiotic fungal community composition and diversity (Johnson et al. 2003; Bever et al. 1996). Equally, it has been proposed that it is the AM fungal diversity that regulates host plant diversity (van der Heijden 2002). In the field, highly diverse plant communities may support a low number of AM fungi, but on the other hand, a low number of hosts can be associated with high AM fungal richness (Johnson and Wedin 1997; Rosendahl and Stukenbrock 2004; Johnson et al. 2010). It also seems possible that the host plant and AM fungal diversities are not strongly linked in real ecosystems, but that both are controlled by a common mediator such as climate, soil factors or history (Jansa et al. 2014; Hazard et al. 2013).

The AM spore population was dominated by members of the Acaulosporaceae (over 95% of all spores in the control soil). The most abundant species in the control soil, *Acaulospora undulata*, represented over half of the total AM spore abundance. High dominance by a single taxon is characteristic of AM fungal communities. For example, across over 30 studies, a single taxon represented 40% of the abundance in AM fungal communities (Dumbrell et al. 2010b). In contrast to our results, spores of the genus *Acaulospora* are reported to be rare in high-altitude soils in the Alps (Oehl et al. 2003) and in high-altitude sites in Tibet (Liu et al. 2011). Intensive land use may result in loss of sensitive taxa (Verbruggen et al. 2012). In the present experiment, removing vegetation and monoculturing resulted in a reduction of Acaulosporaceae spore abundance in particular, which supports the view that Acaulosporaceae are sensitive to disturbance.

### AM spore persistence

Most of the AM fungus morphospecies were present in the soil after 6 years without host plants suggesting that they all form a persistent spore bank. This result is further supported by the fact that monoculturing *Solidago* did not result in loss of any AM fungal species. In a previous study by us, a large portion of the spores was viable after storing for 10 years at − 20 °C (Varga et al. 2015). In the present study, plant colonisation rate in the greenhouse did not differ between control field soil and soil maintained for 6 years without plant cover, indicating that a significant proportion of the AM spores in the no-plant soil were still infective. The ability of AM fungi to persist for 6 years in soil under low Arctic field conditions indicates that the AM fungal population is resilient to environmental perturbations.

We propose that AM fungus spore longevity could be considered a life history trait similar to seed longevity (e.g. Rees 1996) although not included previously in studies of AM fungal life history traits (e.g. IJdo et al. 2010, Chagnon et al. 2013, but see Hart et al. 2001). Comparison of the control and no-plant soils reveals species-specific differences in spore longevity. The abundance of the rare species was not affected after 6 years without a host plant (Table 1). This suggests an ecological AM fungal strategy where low spore production is associated with high longevity of the spores in the spore bank and, vice versa, high spore production with lower longevity of the spores. These characteristics are classic r and K strategy features where production of few high-quality offspring is denoted as a K strategy and many lower quality offspring as an r strategy (Pianka 1970). This assumes that spores that remain long in the spore bank are more costly to produce than those of shorter persistence. Endurance in the spore bank could require an elevated fat content, high allocation of resources to a thick spore wall and other specific spore structures that aid long-term survival. For instance, AM spores are rich in fatty acids (Graham et al. 1995), and high unsaturation may improve survival under fluctuating temperature (Robinson 2001), although it is metabolically costly because unsaturation increases the number of necessary biochemical reactions.

### Effect of vegetation cover on AM fungi

Variation in AM fungal interaction strength and AM fungal richness is of great importance because of the intricate relationship between species diversity, interaction strength and ecosystem stability (Mougi and Kondoh 2012). AM fungal species may interact with each other negatively or positively (Burrows and Pfleger 2002). AM fungal species may positively interact through beneficial effects on a shared host plant (Chen et al. 2017). AM fungi also may negatively interact through competing for resources from a shared host (Pearson et al. 1993). In our *Solidago* monoculture, the dominant AM fungal taxa shared the only host available but had neutral effect on the abundance of other AM fungal species. In contrast, in the controls with diverse host plant communities, the spore network was composed of many positive interactions among the AM fungi. However, one cannot simply assume that the positive interaction networks in the control system are exclusively due to positive effects between AM fungi through a shared host. Direct facilitative interactions among plant species are common under harsh environmental conditions such as in the Arctic (Callaway et al. 2002). It is therefore possible that positive plant–plant interactions contributed to the positive links between AM fungi. This notion is supported by the monoculture AM fungal network in which the dominant AM taxa did not link to each other, suggesting that the positive links between dominants likely resulted from high plant diversity in the control plots.

### Resource acquisition by plants

Control soil supported the greatest resource acquisition by all three plant species. Plant N acquisition was not related to AM fungal community composition in the control soil; instead, it was related to mycorrhizal colonisation intensity. Positive relationships between AM fungal colonisation rate and plant N acquisition have been reported for a wide range of environmental conditions in a grassland (Garcia and Mendoza 2008). Soil organic matter explained plant N content significantly, and it is likely that, in addition to nitrogen, it also contributed to acquisition of other nutrients not measured in this study. The *Anthoxanthum* grass acquired significantly more N but had lower AM fungal colonisation rates than the two herbs. This is in line with grasses having a high capacity for N uptake and use (Silvertown et al. 2010) and hosting lower colonisation than herbs (Wilson and Hartnett 1998). These results suggest that grasses are less responsive to AM fungi than herbs and that environmental perturbations (exemplified here as no-plant and monoculture treatments) that reduce AM spore populations may result in grass-dominated vegetation.

In contrast to the control treatment, AM fungal community composition was a significant factor influencing plant N acquisition in monoculture soil in the greenhouse. Monoculture and no-plant soils had similar soil chemical properties; therefore, any difference between these is likely due to monoculturing *Solidago.* Comparing the response of the three plant species to monoculture and no-plant soils, monoculture soil had a positive effect on *Solidago* N acquisition, but not on the other two species. The positive effect of *Solidago* monoculture on *Solidago* resource acquisition was opposite to what we expected based on frequently reported negative plant-soil feedback (e.g. Bonanomi et al. 2005; Kempel et al. 2018). Monoculturing *Solidago* under field conditions changed the soil spore community which became dominated by *Glomus hoi*. Monoculturing previously has been shown to change AM fungal communities in agricultural fields (Oehl et al. 2003) and in experimental meadow systems (Burrows and Pfleger 2002). The frequency of arbuscules in the *Solidago* test plants was also higher in the monoculture soils than when grown in the no-plant soils. Frequency of arbuscules is considered an indicator of the exchange of resources between the host and the fungus (Saito 2000). The higher frequency of arbuscules did not increase the *Solidago* host benefit measured in terms of growth, but resulted in higher host N concentration. AM fungi have been shown to be important in plant N acquisition (Govindarajulu et al. 2005; Whiteside et al. 2012) and plant nitrogen concentration and AM fungal colonisation rates correlated significantly in the present study. Host plants have been shown to reward preferentially the most beneficial AM fungi under greenhouse conditions (Werner and Kiers 2015), which should lead to an increase in host-beneficial AM fungal species. The presently observed positive plant-soil feedback is consistent with the view that the host plant selects beneficial AM fungal symbionts under field conditions.

## Conclusions

Altogether, these results suggest that restoration efforts and other human activities where single or a few plant species are introduced to keep the soil surface vegetated (Gómez-Aparicio 2009) result in selective AM fungal communities. Changes in AM community are pivotal because the AM fungal community composition is important in seedling establishment (Koorem et al. 2012) and affects the plant community composition and the successional trajectory of the ecosystem (Koziol and Bever 2016). The AM spore bank is resistant to short-term disturbance and consequently buffers changes in vegetation cover for a few years at least. AM fungi have species-specific persistence rates in soil, and AM spore longevity could be considered a life history trait. This work suggests a testable hypothesis of selection for long AM spore persistence in disturbed ecosystems versus short-term AM spore persistence in stable ecosystems.
